# Controversies in the management of craniosynostosis at an advanced age

**DOI:** 10.1186/2045-8118-12-S1-P26

**Published:** 2015-09-18

**Authors:** Petra M Klinge, Rajiv Iyengar, Stephen Sullivan, Wendy Chen, Jerrold Boxerman, Helena Taylor

**Affiliations:** 1 Department of Neurosurgery, Rhode Island Hospital, USA; 2 Department of Plastic Surgery, Rhode Island Hospital, USA; 3 Department of Plastic Surgery, Rhode Island Hospital, USA; 4 Department of Pediatric Ophthalmology, Rhode Island Hospital, USA; 5 Department of Neuroradiology, Rhode Island Hospital, USA; 6 Department of Plastic Surgery, Rhode Island Hospital, USA

## Introduction

In this study, we describe a cohort of patients with a delayed presentation of non-syndromic craniosynostosis.

## Methods and patients

10 consecutive patients with delayed diagnosis and treatment of craniosynostosis between January 2008 and February 2015 were included. Inclusion criteria were age greater than two years at time of initial evaluation, fusion of at least one suture, and adequate imaging studies including CT or MRI or both. All children were evaluated by a multidisciplinary team including a craniofacial plastic surgeon, neurosurgeon, ophthalmologist, neuroradiologist, developmental specialist and medical geneticist. Either intraparenchymal ICP monitoring was performed for 24-48 hours or intraoperative epidural ICP monitoring at the time of surgery.

## Results

Headaches were improved in all patients and the most reliable symptom. The comparative perioperative data (median estimated blood loss, operative time and hospital stay) between reconstructions on infants versus older children Delayed surgery required greater median operative time, but median blood loss and duration of hospital stay were comparable. There were no complications (e.g. return to OR, prolonged intubation) in either this cohort or the infant controls.

## Conclusion

While the debate rages over the functional utility of cranial vault remodeling for non-syndromic craniosynostosis, this series suggests a high rate of morbidity in neglected cases. Seven out of ten children had documented developmental delay, five out of ten had Chiari malformations, and five out of ten had debilitating headaches. Three presented with documented ICP elevation. This series suggests that delaying cranial vault reconstruction for craniosynostosis is associated with a high incidence of developmental delay, headaches and coincident Chiari malformations. Delayed reconstruction and cranial vault expansion can be performed for these older patients with low morbidity but added operative time.

